# IFI16 Preferentially Binds to DNA with Quadruplex Structure and Enhances DNA Quadruplex Formation

**DOI:** 10.1371/journal.pone.0157156

**Published:** 2016-06-09

**Authors:** Lucia Hároníková, Jan Coufal, Iva Kejnovská, Eva B. Jagelská, Miroslav Fojta, Petra Dvořáková, Petr Muller, Borivoj Vojtesek, Václav Brázda

**Affiliations:** 1 Institute of Biophysics, Academy of Sciences of the Czech Republic, Královopolská 135, 612 65, Brno, Czech Republic; 2 Department of Biochemistry, Faculty of Science, Masaryk University, Kotlarska 2, 61137, Brno, Czech Republic; 3 RECAMO, Masaryk Memorial Cancer Institute, Zluty kopec 7, 656 53, Brno, Czech Republic; University of Quebec at Trois-Rivieres, CANADA

## Abstract

Interferon-inducible protein 16 (IFI16) is a member of the HIN-200 protein family, containing two HIN domains and one PYRIN domain. IFI16 acts as a sensor of viral and bacterial DNA and is important for innate immune responses. IFI16 binds DNA and binding has been described to be DNA length-dependent, but a preference for supercoiled DNA has also been demonstrated. Here we report a specific preference of IFI16 for binding to quadruplex DNA compared to other DNA structures. IFI16 binds to quadruplex DNA with significantly higher affinity than to the same sequence in double stranded DNA. By circular dichroism (CD) spectroscopy we also demonstrated the ability of IFI16 to stabilize quadruplex structures with quadruplex-forming oligonucleotides derived from human telomere (HTEL) sequences and the *MYC* promotor. A novel H/D exchange mass spectrometry approach was developed to assess protein interactions with quadruplex DNA. Quadruplex DNA changed the IFI16 deuteration profile in parts of the PYRIN domain (aa 0–80) and in structurally identical parts of both HIN domains (aa 271–302 and aa 586–617) compared to single stranded or double stranded DNAs, supporting the preferential affinity of IFI16 for structured DNA. Our results reveal the importance of quadruplex DNA structure in IFI16 binding and improve our understanding of how IFI16 senses DNA. IFI16 selectivity for quadruplex structure provides a mechanistic framework for IFI16 in immunity and cellular processes including DNA damage responses and cell proliferation.

## Introduction

IFI16 (interferon-inducible protein 16) has multiple biological functions; it is a DNA sensor important in inflammasome activation [1, 2], but it also plays roles in transcriptional regulation [3, 4] and cell proliferation [5]. IFI16 belongs to the highly homologous HIN-200 (hemopoietic expression—interferon-inducibility—nuclear localization) protein family characterized by a 200 amino acid motif containing a DNA binding domain at the C-terminus and a PYRIN domain at the N-terminus, involved mainly in protein-protein interactions. The human HIN-200 family is composed of four characterized members; absent in melanoma 2 (AIM2), interferon-inducible protein X (IFIX), myeloid cell nuclear differentiation antigen (MNDA) and IFI16 [6, 7]. IFI16 differs from other members by the presence of two HIN domains [7] and was detected not only in the nucleus, but also in the cytoplasm [8, 9]. IFI16 subcellular localization is influenced by the cell type [10], post-translational modification [11, 12] and cell treatment. For example, pathogen invasion causes the formation of IFI16 foci in the cytoplasm and induces interferon β (*IFNB*) gene expression [9] and UV-light causes the transfer of IFI16 from the nucleus to the cytoplasm [13].

IFI16 cooperates with other proteins in transcriptional regulation and DNA repair. Binding of IFI16 HIN-A domain to the C-terminus of p53 results in enhanced DNA binding of p53 and increased transcriptional activation of p21 [14]. Moreover, IFI16 is involved in the p53-mediated pathway and DNA damage recognition through breast cancer-associated protein-1 (BRCA1) interaction, where BRCA1 relocates IFI16 from the cytoplasm to the nucleus and IFI16 is necessary for full activation of DNA repair after ionizing radiation [15–17]. As a DNA sensor, IFI16 stimulates the formation of inflammasomes in certain cell types during infection with Kaposi Sarcoma-associated herpesvirus [1, 2], Herpes simplex virus 1 [18], Epstein-Barr virus [19] and Human immunodeficiency virus (HIV-1) [20]. The DNA sensing ability of IFI16 is also related to the activation of interferon β expression through interaction with stimulator of interferon genes [9], and interferon α expression [4].

IFI16 was first identified as a DNA binding protein by Dawson and Trapani in 1995 [21]. In 2008, IFI16 HIN-A was described as an RPA-like protein, sharing the same oligonucleotide / oligosaccharide domain and preference for single stranded DNA over double stranded DNA [22]. According to Unterholzner et al., IFI16 binding to DNA is not sequence-specific or AT content-dependent, but is strongly DNA length-dependent [9]. Based on crystallographic studies, the IFI16 HIN-B—double stranded DNA interface is accomplished through electrostatic interactions between the negatively charged sugar-phosphate backbone and positively charged protein residues [23]. Based on structural analysis and binding experiments of the HIN-A and HIN-B domains with double stranded DNA, a model of non-interacting beads on a string was proposed [23, 24]. In a recent study, the single stranded DNA preference was questioned for the full length wild type protein and DNA-length dependence was characterized in more detail, revealing cooperative assembly of IFI16 filaments on double stranded DNA [25]. IFI16 binding to long plasmid DNA was studied and preferences for supercoiled over linear forms and for cruciform structure over double stranded DNA was observed [26].

Since the description of double-stranded B-DNA, the knowledge of variability of DNA structures has greatly expanded. The presence of cruciform, triplex and quadruplex structures was demonstrated by many techniques *in vitro*. Nowadays, there is substantial evidence for the presence of these unusual structures *in vivo* and their significance is being uncovered [27, 28]. Large numbers of potential quadruplex sequences were predicted by *in silico* analysis [29]. To date, many quadruplex DNA sequences in the human genome were characterized, for example in repetitive G-rich sequences such as telomeres [30] and in the promoters of oncogenes such as *MYC* [31], *KIT* [32], *BCL2* [33] and *TERT* [34]. The transition from double stranded DNA to quadruplex structure influences processes related to cancer through expression of target genes [35–37] or through inhibition of telomerase processivity [38]. Recently, quadruplex DNA and RNA structures have been detected in viral genomes, notably Epstein-Barr virus [39], HIV-1 [40, 41] and human papillomaviruses [42]. Contemporary results show the importance of quadruplex structure in maintaining chromosome integrity, replication, regulation of transcription and translation [43].

Here, we demonstrate that IFI16 shows preferential binding to quadruplex DNA with positive effects on quadruplex DNA formation and stabilization. Our findings provide more insight into IFI16 DNA binding and the connection between IFI16 and quadruplexes as biologically active DNA structures adds a new dimension to our understanding of the roles of IFI16.

## Materials and Methods

### DNA

Supercoiled plasmid DNAs of pBluescript II SK (-) and the derived plasmid pCMYC were isolated from *DH5α* as described in the QIAGEN protocol (QIAGEN GmbH, Hilden, Germany). pCMYC plasmid (containing 141 bp of nuclease hypersensitive element III1a (NHEIII) region of the human *MYC* promoter forming G-quadruplex) was constructed by cloning the 141 bp *Eco*RI/*Hind*III restriction fragment of pNHE III_1a_ plasmid [44] into the *Eco*RI/*Hind*III site of pBluescript and was kindly provided by Dr. Marie Brazdova. Plasmids were linearized by *Eco*RI restriction enzyme (New England Biolabs, Ipswich, MA, USA).

### Synthetic oligonucleotides

Synthetic oligonucleotides with and without FAM-3’-end labeling were purchased from Integrated DNA Technologies, Inc., Coralville, IA, USA. The oligonucleotide sequences of single stranded, double stranded, cruciform and quadruplex DNA are shown in Table 1. Complementary oligonucleotides for double stranded and cruciform structure were annealed by incubation at 95°C for 5 min with subsequent cooling to room temperature. Oligonucleotide for quadruplex formation was heated to 95°C in TE buffer and then incubated with 50 mM KCl at room temperature for 16 h.

**Table 1:** DNA sequences of oligonucleotides.

name	structure	5´-3´sequence
SS A50	SS	AAAAAAAAAAAAAAAAAAAAAAAAAAAAAAAAAAAAAAAAAAAAAAAAAA
CF	CF	GAATTCAGCACGAGTCCTAACGCCAGATCT
		AGATCTGGCGTTAGGTGATACCGATGCATC
		CACTAGTCGTAAGCCACTCGTGCTGAATTC
		CATGCATCGGTATCAGGCTTACGACTAGTG
Q HTEL		GGGTTAGGGTTAGGGTTAGGGTTAGGGTTAGGGTTAGGGTTAGGGTTAGGG
DS HTEL	DS	GGGTTAGGGTTAGGGTTAGGGTTAGGGTTAGGGTTAGGGTTAGGGTTAGGG
		CCCTAACCCTAACCCTAACCCTAACCCTAACCCTAACCCTAACCCTAACCC
Q NHEIII	Q	TTGGGGCGCTTAT**GGGG**AGGGT**GGGG**AGGGT**GGGG**AAGGT**GGGG**AGGAGACT
DS NHEIII	DS	TTGGGGCGCTTATGGGGAGGGTGGGGAGGGTGGGGAAGGTGGGGAGGAGACT
		AGTCTCCTCCCCACCTTCCCCACCCTCCCCACCCTCCCCATAAGCGCCCCAA

### Protein purification

Full length *IFI16* gene was PCR amplified from human IFI16 cDNA and subcloned into the pET15b expression vector (Novagen, Merck KGaA, Darmstadt, Germany). The sequence of the resulting expression clone was verified. Protein with N-terminal 6xHis-tag was expressed in *E*. *coli* BL21-CodonPlus cells (Stratagene, Agilent, Santa Clara, CA, USA) and purified by affinity chromatography (TALON resin, Clontech Laboratories, Inc., Mountain view, CA, USA). After elution proteins were gel filtrated.

### Gel electrophoretic mobility shift assays on native PAGE

Labelled oligonucleotides (5 pmol) and IFI16 protein were mixed at different molar ratios (1:0 / 1:0.25 / 1:0.5 / 1:1 / 1:2 / 1:4 / 1:8) in 15 μl DNA binding buffer, incubated for 15 min at 4°C and loaded onto non-denaturing polyacrylamide gels with 4% top and 16% bottom layer containing 0.33x Tris-borate-EDTA buffer, 50 mM KCl. Electrophoresis was performed for 3h at 50 V at 4°C. The gels were visualized on a LAS-3000 image analyzer (Fujifilm) by Blue LED (460nm) incident light source and processed digitally.

### Gel electrophoretic mobility shift assays on agarose gel

DNA (100 ng) and IFI16 were mixed at increasing molar ratios in 10 μl of DNA binding buffer (5 mM Tris-HCl, pH 7.0, 1 mM EDTA, 50 mM KCl, 0.01% Triton X-100). After 15 min incubation at 4°C samples were loaded onto a 1% agarose gel containing 0.33x Tris-borate-EDTA buffer. Agarose electrophoresis was performed for 3 h at 100 V (usually 4 V/cm) at 4°C. The gels were stained with ethidium bromide and photographed.

### CD spectroscopy

CD measurements were carried out in a Jasco 815 (Jasco International Co., Ltd.,Tokyo, Japan) dichrograph in 1 cm path-length quartz Hellma microcells placed in a thermostatically regulated cell holder at 23°C. A set of four scans was averaged for each sample with a data pitch of 0.5 nm and 100 nm.min^-1^ scan speed. CD signal was expressed as the difference in the molar absorption, Δε of the left- and right-handed circularly polarized light, molarity being related to DNA strands. Precise DNA strand concentrations were determined on the basis of UV absorption at 260 nm measured in TE buffer pH 8, using molar extinction coefficients of 539,600, and 541,400 M^-1^cm^-1^ calculated according Gray et al [45] for HTEL and NHEIII sequences respectively. Experimental conditions were changed directly in the cells by adding solution (KCl protein buffer: 50 mM KCl, 5 mM Tris/HCl pH 7.6, 10% glycerol, 2 mM DTT, 0.1 mM EDTA; NaCl protein buffer: 20 mM HEPES, pH 7.6, 500 mM NaCl, 10% glycerol, 2 mM DTT) with or without the protein and the final DNA strand concentration was corrected according to the increase in volume.

### H/D exchange—Sample preparation

At first a sample for peptide mapping was prepared to obtain the protein coverage. IFI16 protein (1 μM) was dissolved in 1% DMSO and the pH adjusted using 0.88 M HCl in 1 M glycine. The next step was preparation of deuterated samples. IFI16 protein was incubated with DNA (single stranded, double stranded NHEIII, quadruplex NHEIII) for 5 min, then the protein-DNA complex was initiated for deuteration by dilution into 1% DMSO in deuterated water containing 5 mM Tris/HCl, 0.1 mM EDTA, 50 mM KCl, pH 7.6 to stabilize DNA. The H/D exchange was carried out at 4°C and was quenched by the addition of 0.88 M HCl in 1 M glycine after 15 min. Then 3 μg of pepsin was added and the protein was digested at 4°C. After 2 min the sample was placed on a strong anionic exchange column (Q-sepharose). Peptides were spun through the column (1 min, 8000 rcf) while DNA was captured on the column. Finally, the sample was rapidly frozen in liquid nitrogen. Simultaneously a control sample was prepared, where the protein was incubated with 1% DMSO (instead of DNA).

### H/D exchange—Sample measurement and data processing

Each sample was thawed and injected onto an immobilized pepsin column (66 μl bed volume, flow rate 20 μl/min, 2% acetonitrile / 0.05% trifluoroacetic acid). Peptides were trapped and desalted on-line on a peptide microtrap (Michrom Bioresources, Auburn, CA, USA) for 2 min at flow rate 20 μl/min. Next, the peptides were eluted onto an analytical column (Jupiter C18, 1.0 x 50 mm, 5 μm, 300Å, Phenomenex, Torrance, CA, USA) and separated using a linear gradient elution of 10% B in 2 min, followed by 31 min isocratic elution at 40% B. Solvents were: A– 0.1% formic acid in water, B– 80% acetonitrile / 0.08% formic acid. The immobilized pepsin column, trap cartridge and the analytical column were kept at 1°C. Mass spectrometric analysis was carried out using an Orbitrap Elite mass spectrometer (Thermo Fisher Scientific, Whaltan, MA, USA) with ESI ionization on-line connected with a robotic system based on the HTS-XT platform (CTC Analytics company, Zwingen, Switzerland). The instrument was operated in a data-dependent mode for peptide mapping (LC-MS/MS). Each MS scan was followed by MS/MS scans of the top three most intensive ions from both CID and HCD fragmentation spectra. Tandem mass spectra were searched using SequestHT against the cRap protein database (ftp://ftp.thegpm.org/fasta/cRAP) containing the IFI16 protein sequence. Sequence coverage was analysed with Proteome Discoverer 1.4 software (Thermo Fisher Scientific). Analysis of deuterated samples was performed in LC-MS mode with ion detection in the orbital ion trap and the data were processed using HD Examiner (Sierra Analytics, Modesto, CA, USA).

## Results

### Recognition of quadruplex structures by IFI16 in plasmid DNA

**Fig 1:**
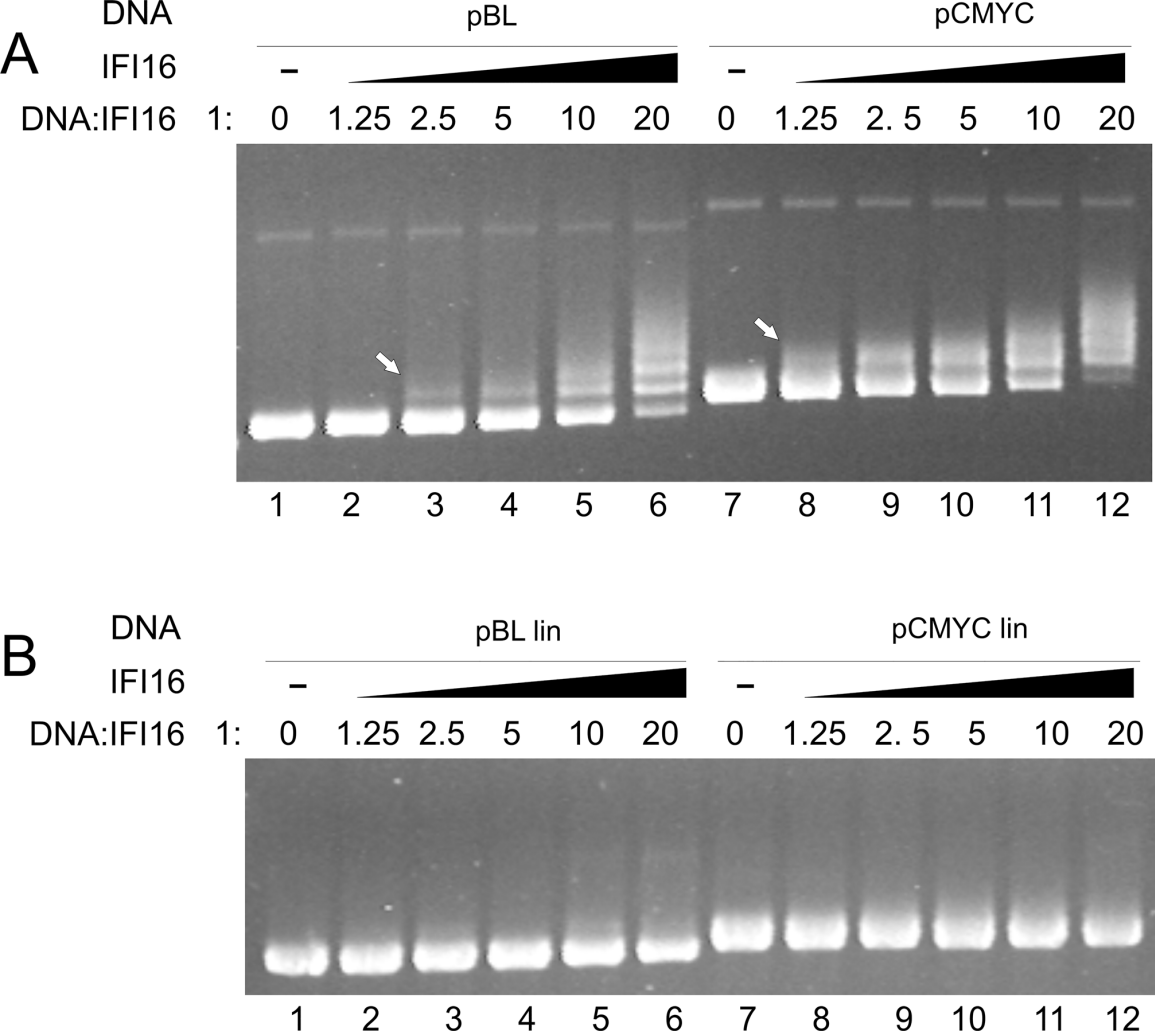
Binding of IFI16 protein to supercoiled DNAs. (A) 100 ng sc pBluescript (lane 1–6) and sc pCMYC (lane 7–12) were incubated with increasing concentrations of IFI16 (molar ratio DNA:protein 1:0 / 1:1.25 / 1:2.5 / 1:5/ 1:10 / 1:20) in binding buffer (5 mM Tris-HCl, pH 7.0; 1 mM EDTA, 50 mM KCl and 0.01% Triton X-100) on ice for 15 min. The electrophoresis ran for 3 h at 100 V at 4°C. (B) 100 ng linear pBluescript (lane 1–6) and linear pCMYC (lane 7–12) were incubated with increasing concentrations of IFI16 (molar ratio DNA: protein 1:0 / 1:1.25 / 1:2.5 / 1:5/ 1:10 / 1:20) in binding buffer (5 mM Tris-HCl, pH 7.0; 1 mM EDTA, 50 mM KCl and 0.01% Triton X-100) on ice for 15 min. The electrophoresis ran for 3 h at 100 V at 4°C.

To compare IFI16 binding to quadruplex and double stranded DNAs derived from the *MYC* promoter we used electrophoretic mobility shift assay with DNA plasmids on agarose gel. It was previously demonstrated that IFI16 binds preferentially to supercoiled DNA compared to the linear form of the same plasmid DNA [29]. Moreover, IFI16 was described as a length-dependent DNA binding protein [22, 28]. Considering these observations, we were interested in whether IFI16 is capable of recognizing quadruplex structures stabilized as a local structure in large negatively supercoiled DNA molecules (where they represent only a small portion of a DNA substrate which per se is relatively strongly bound by the protein). In this study we used two supercoiled plasmids: pBluescript was used as a model of supercoiled DNA without quadruplex structure and pCMYC containing 141 bp from the NHEIII region of *MYC* promoter that includes G:C-rich sequence was used as a model for binding to quadruplex DNA. Formation of the quadruplex structure in the plasmid was induced by negative supercoiling (due to destabilization of duplex DNA, thus favoring separation of strands and folding of the G-rich strand into the quadruplex). Presence of the quadruplex, featuring an open non-B structure in the plasmid, was confirmed by S1 nuclease cleavage as described earlier for plasmids with cruciform structure [46]. In addition, probabilities of quadruplex formation in both plasmids were analyzed by the free software QGRS Mapper [47] and only pCMYC (but not pBluescript) showed possible formation of one predicted G-quadruplex for 4 minimal G-group size, in agreement with expectations (Table 1, underlined G-quartets in the quadruplex NHEIII sequence are predicted to form quadruplex structure in pCMYC). Various amounts of IFI16 protein were incubated with 100 ng of plasmid DNAs and the resulting complexes were then separated on 1% agarose gels. In Fig 1A, lanes 1 and 7, free DNAs without IFI16 protein were loaded. After addition of IFI16 (molar ratio protein: DNA 1.25:1 (lane 2 and 8), 2.5:1 (lane 3 and 9), 5:1 (lane 4 and 10), 10:1 (lane 5 and 11) 20:1 (lane 7 and 12)) we observed different band patterns due to IFI16 DNA binding. The binding of IFI16 to DNA was visible as shifted (retarded) band(s) and/or as a decrease of the free DNA band intensity (decreasing to total loss caused by saturation of protein binding). While we observed retarded band(s) of pBluescript from molar ratio 2.5:1 (Fig 1A, lane 3), pCMYC was evidently bound from the lowest protein concentration tested (molar ratio1.25:1, Fig 1A, lane 8). At higher protein concentration (lane 6) pBluescript-IFI16 complexes formed multiple shifted bands but the free form of pBluescript was still visible even at a protein:DNA ratio of 20:1, whereas pCMYC (lane 12) was completely bound with IFI16 protein under the latter conditions and free pCMYC was not observed. Strikingly, for protein:DNA ratios 1:1.25–1:5 pCMYC formed a single strong retarded band (compared to pBluescript forming multiple weak bands), suggesting a single strongly preferred protein-DNA complex formed at a specific site. Similar behavior was previously observed with a plasmid containing a single target site for p53 upon formation of specific p53-DNA complexes [48]. In pCMYC, such a preferred site for IFI16 binding can be the quadruplex structure. In contrast, pBluescript contains no preferentially bound site and at higher protein-DNA ratios it forms IFI16-DNA complexes with various stoichiometries (again, in analogy with earlier observed p53-DNA binding -[48]). Hence, our results strongly suggest selective binding of IFI16 to a quadruplex existing as a local supercoil-stabilized structure in plasmid DNA i.e., with structural arrangement more complex than represented by short oligonucleotide targets. In Fig 1B we compared the binding of IFI16 to the linear forms of both tested plasmids. In contrast to the above described results with scDNAs, structurally unconstrained linearized forms of the same plasmid did not apparently bind IFI16 at protein-DNA ratios between 1.25:1 and 20:1 (Fig 1B).

### Stabilization of quadruplex structure by IFI16

**Fig 2:**
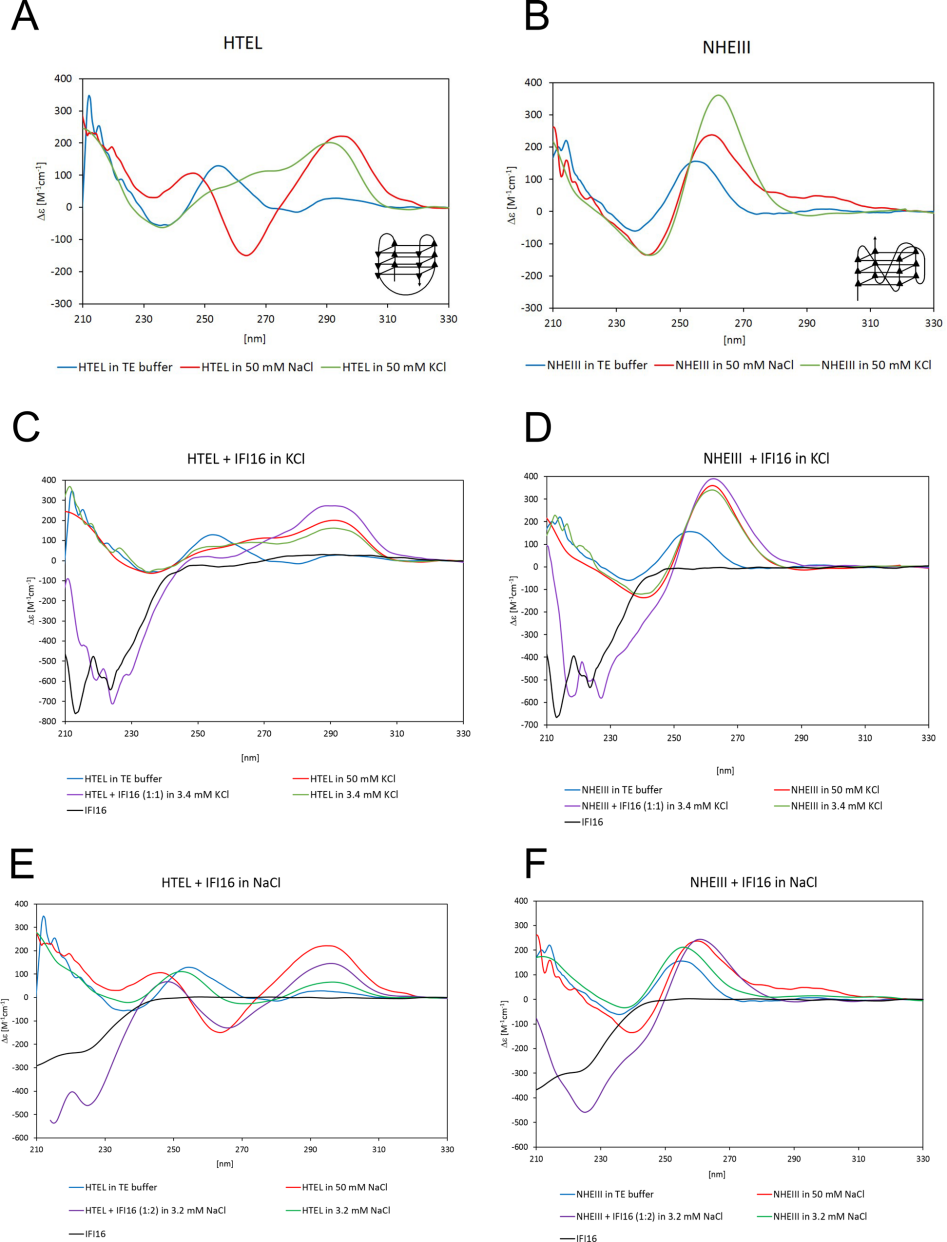
CD spectroscopy of quadruplexes and their stabilization by IFI16. (A) CD spectra of oligonucleotide HTEL in TE buffer after denaturation (blue line), in TE buffer + 50 mM NaCl (red line) and in TE buffer + 50 mM KCl (green line). (B) CD spectra of oligonucleotide NHEIII in TE buffer after denaturation (blue line), in TE buffer + 50 mM NaCl (red line) and in TE buffer + 50 mM KCl (green line). The schematic drawings represent quadruplex structures of HTEL and NHEIII sequences. (C) The effect of recombinant IFI16 on HTEL quadruplex formation in potassium ions. CD spectra description: HTEL oligonucleotide in TE buffer (blue line), HTEL in TE buffer with 50 mM KCl (red line), HTEL in TE buffer + protein buffer with final concentration 3.4 mM KCl (green line), HTEL in TE buffer + IFI16 in protein buffer at molar ratio 1:1 and final concentration 3.4 mM KCl (violet line), IFI16 protein in protein buffer with final concentration 3.4 mM KCl in TE buffer (black line). (D) The effect of recombinant IFI16 on NHEIII quadruplex formation in potassium ions. The same description of curves as in C (NHEIII instead of HTEL). (E) The effect of recombinant IFI16 on HTEL quadruplex formation in sodium ions. CD spectra description: HTEL oligonucleotide in TE buffer (blue line), HTEL in TE buffer with 50 mM NaCl (red line), HTEL in TE buffer + protein buffer with final concentration 3.2 mM NaCl (green line), HTEL in TE buffer + IFI16 in protein buffer at molar ratio 1:2 and final concentration 3.2 mM NaCl (violet line), IFI16 protein in protein buffer with final concentration 3.2 mM NaCl in TE buffer (black line). (F) The effect of recombinant IFI16 on NHEIII quadruplex formation in sodium ions. The same description of curves as in E (NHEIII instead of HTEL).

Many quadruplex binding proteins were described recently [28]. Some quadruplex binding proteins resolve these structures, while others induce and enhance quadruplex formation. Having provided initial evidence that IFI16 recognizes quadruplex structure in plasmid DNA, we validated binding to two short quadruplexes and further investigated the effect of IFI16 protein on the formation and stabilization of quadruplex structures by CD spectroscopy. We used the HTEL oligonucleotide which forms an antiparallel (2+2) quadruplex structure and the NHEIII oligonucleotide which folds into a parallel quadruplex [49–51]. The HTEL oligonucleotide CD spectra in the presence of 50 mM KCl and 50 mM NaCl are shown at Fig 2A—the unstructured oligonucleotide HTEL (blue line) is represented by the peak at 255 nm, the quadruplex structure is demonstrated by formation of a peak at 296 nm typical for antiparallel (2+2) quadruplex structure. The NHEIII oligonucleotide folds into a parallel quadruplex in the presence of 50 mM KCl more efficiently than in the presence of 50 mM NaCl [52] (Fig 2B). The quadruplex structure is demonstrated by peak shift to 260 nm and an increase in height. The differences in structure of unfolded oligonucleotides, oligonucleotides folded to quadruplex parallel or antiparallel structure and double helical oligonucleotides by CD spectroscopy are summarized in Vorlickova et al. [50].

The effect of IFI16 on quadruplex stability was studied in the presence of either of the salts for both quadruplex forming oligonucleotides. First, we measured the CD spectra of the oligonucleotides in TE buffer after denaturation where the CD spectra suggest their unfolded state. Then we added the protein in buffer containing KCl or NaCl to the oligonucleotide in TE buffer. The same volume of protein buffer was added to the unfolded oligonucleotide as a control to see the effect of protein buffer itself on DNA structure. CD spectra indicating the IFI16 stabilization effect are shown in Fig 2C–2F. The unstructured oligonucleotide HTEL (Fig 2C) in TE buffer (blue line) is represented by the peak at 255 nm. After addition of the protein-free buffer (3.4 mM KCl in final volume) (green line), the initiation of formation of the quadruplex structure is visible as peaks appearing at 296 nm and 264 nm, and a decrease of the 255 nm peak. The addition of IFI16 (in molar ratio IFI16:oligonucleotide 1:1) to the unfolded oligonucleotide causes stronger quadruplex formation (magenta line), surprisingly even stronger than in the presence of 50 mM KCl (red line). Hence, IFI16 stimulates and stabilizes quadruplex structure formation. The short wavelength part of the spectrum is influenced by absorption of protein (black line for IFI16 without oligonucleotide).

The same experiment was performed with the protein dissolved in buffer containing NaCl (Fig 2E). The CD spectra are colored as in Fig 2C. CD spectrum of HTEL in 50 mM NaCl is characterized by a maximum at 296 nm, similar to that observed in the presence of potassium ions (for comparison of the spectra see Fig 2A) and a minimum at 264 nm. At low NaCl concentrations (3.2 mM NaCl corresponding to the salt concentration after protein addition) there is only a small increase at 296 nm in the CD spectrum. IFI16 addition induced a larger change in the CD spectrum shape compared to the effect of 50 mM NaCl in the absence of the protein. Quadruplex formation in the presence of sodium ions (without IFI16) required higher salt concentration than observed for potassium ions. For this reason, molar ratio 1:2 (DNA:protein) was used (ratio 1:1 was too low to induce quadruplex formation in 1.6 mM NaCl, not shown). In the presence of protein, synergic effects of potassium or sodium ions and IFI16 on quadruplex formation were observed. Similarly, the unfolded oligonucleotide NHEIII spectrum (Fig 2D, blue line) showed a characteristic peak around 253 nm. Addition of protein-free buffer containing 3.4 mM KCl (green line) caused a shift to 260 nm and an increase in height, suggesting formation of the parallel quadruplex structure. The addition of IFI16 in the same buffer containing 3.4 mM KCl in the final volume (magenta line) increased the peak height more than addition of buffer alone (green line). The stabilization effect of IFI16 was even stronger than that of 50 mM KCl (red line). The stabilization effect of IFI16 on NHEIII quadruplex structure was less visible because the presence of 3.4 mM KCl (without protein) induced quadruplex formation. NHEIII quadruplex formation in sodium ions is less effective (the signature quadruplex peak was at 260 nm and was smaller in 50 mM NaCl than in 50 mM KCl, for comparison see Fig 2B). Therefore, the differences in CD spectra with and without protein are considerably larger (Fig 2F). The CD spectrum of NHEIII containing 3.2 mM NaCl (green line) exhibited a maximum at 253 nm, similar to unfolded NHEIII (blue line), and initial quadruplex formation was visible as an increase of the band. IFI16 addition caused a considerable increase in peak height and a shift to 260 nm, comparable to the effect of 50 mM NaCl alone. Again, higher protein amount was used (1:2 NHEIII:IFI16 molar ratio) because low sodium ion concentration was insufficient to support quadruplex formation at the 1:1 protein:DNA ratio. Thus, the synergic effect of ions and protein on quadruplex formation is predicted for all experimental conditions and it appears that IFI16 binds and stabilizes both quadruplexes to a comparable extent No preference for parallel/antiparallel conformation was observed by either CD spectroscopy or EMSA.

### Quadruplex DNA changes IFI16 accessibility as detected by H/D exchange

**Fig 3:**
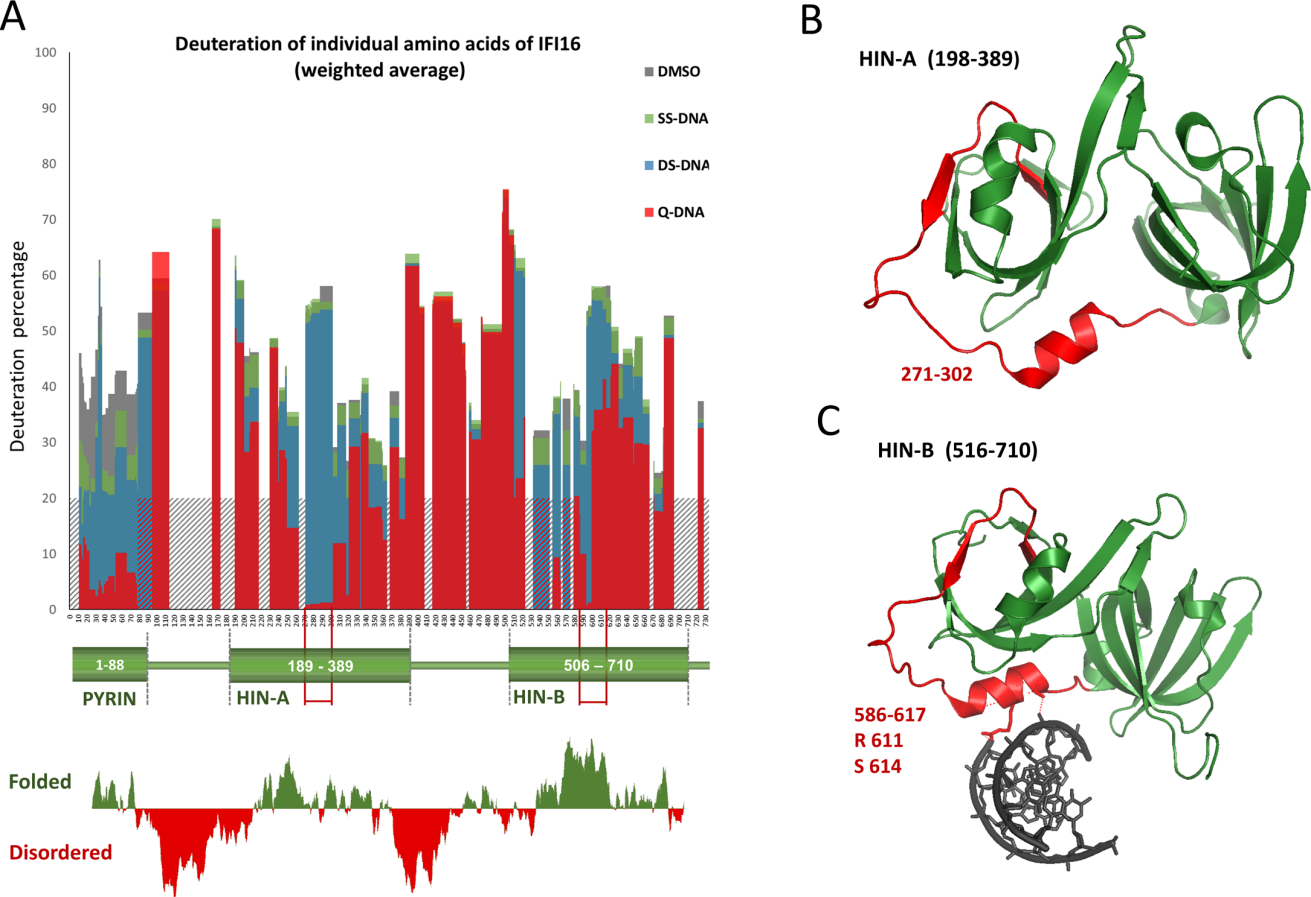
H/D exchange of IFI16 in response to DNA interaction. (A) H/D exchange of IFI16 in response to DNA interaction was analyzed in four reactions: IFI16 protein without DNA as a control, IFI16 with single stranded DNA oligonucleotide (SS DNA), IFI16 with double stranded DNA oligonucleotide (DS NHE III) and IFI16 with DNA forming quadruplex structure (Q NHEIII). H/D exchange was quenched at 900 s after addition of deuterium. The graph shows percentage deuteration of individual amino acids of IFI16 calculated as weighted average of corresponding peptides [53]. Shaded area of the graph shows the areas not covered by peptides. The deuteration spectrum is aligned with the domain structure of IFI16 and with prediction of disordered regions (FoldIndex [54]). (B) Structure of the first HIN-A domain (PDB 2OQ0) corresponding to amino acids 198–389 of IFI16 [14]. (C) Complex of the second HIN-B domain with DNA (PDB 3RNU) corresponding to amino acids 516–710 of IFI16 [23]. In (B) and (C) the helical linker peptide exhibiting the most significant changes in percentage of deuteration in the presence of quadruplex DNA is highlighted in red.

To elucidate which part of the IFI16 protein is involved in binding to quadruplex DNA we used hydrogen deuterium (H/D) exchange mass spectrometry analyses. The structure of full length IFI16 includes both structured domains and primary disordered regions. Fig 3A shows alignment of IFI16 structure with H/D exchange. The grey color shows deuteration of free IFI16 protein, consistent with the predicted structure. The three domains PYRIN, HIN-A and HIN-B exhibited significantly lower H/D exchange in comparison to the primary disordered regions. The experiment also revealed a complex interaction of IFI16 with DNA. Green color shows changes of deuteration of IFI16 protein by interaction with single stranded DNA. We observed only slight changes of deuteration (about 10%) in the N-terminal part of the protein (aa 0–80, corresponding to the PYRIN domain). With double stranded DNA, the change in deuteration was 10–15%. We observed larger changes of IFI16 deuteration with quadruplex DNA (30–35%). While deuteration of the PYRIN domain was decreased by all tested DNAs (in the order single stranded DNA < double stranded DNA < quadruplex DNA), deuteration of both HIN domains was changed only in the presence of quadruplex DNA. We found that all three domains of the protein are simultaneously influenced by interaction with quadruplex DNA, with peaks in parts of the HIN-A (271–302, Fig 3B) and HIN-B (586–617, Fig 3C) domains. Interestingly the biggest changes in deuteration are located in structurally identical regions of HIN-A and HIN-B domains that are closely linked to OB-folds. Fig 3B shows the structure of the HIN-A domain as determined by Liao et al. [14]–the region with the largest changes in deuteration is highlighted in red. Jin et al. [23] showed that amino acids R611 to S614 in the HIN-B domain form polar contacts with DNA (Fig 3C). The same area is not accessible for deuteration after IFI16 binding to quadruplex DNA. However, our results show that protection of the HIN domain by quadruplex DNA covers a much larger area than displayed in the crystal structure 3RNU. Since the region 587–595 exhibits the highest protection in the presence of quadruplex DNA but lacks the interaction with ssDNA in the crystal structure, we can assume that this region is responsible for additional contacts with quadruplex DNA. Likewise, we can propose that other positively charged amino acids such as R601 and K607 form polar contacts with quadruplex DNA. From these results we conclude that preferential binding to quadruplex structure is facilitated by specific and dimensional coordination of these domains.

### IFI16 binds preferentially to natural human quadruplex-forming DNA elements

**Fig 4:**
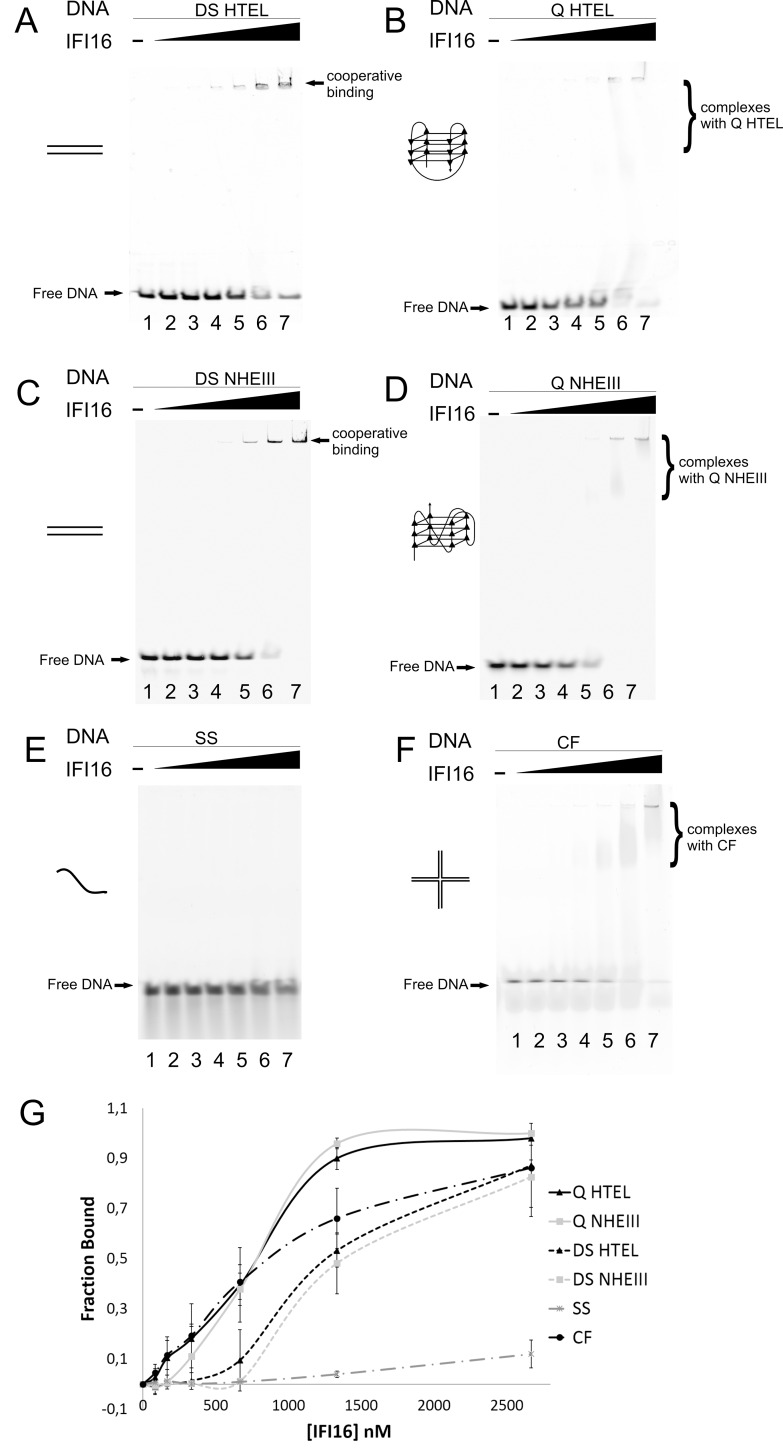
Comparison of IFI16 DNA binding to structurally different DNA targets. EMSA was performed with 5 pmol of labeled oligonucleotides forming DS from human telomere sequence–DS HTEL (A) and G-quadruplex from one strand of the same sequence–Q HTEL (B), DS from NHE III region from *MYC* promoter–DS NHEIII (C) and G-quadruplex from one strand of the same sequence–Q NHEIII (D), SS (E) and cruciform (F) and increasing IFI16 concentrations (0 / 1.25 / 2.5 / 5 / 10 / 20 / 40 pmol), incubated in binding buffer (5 mM Tris-HCl, pH 7.0, 1 mM EDTA, 50 mM KCl and 0.01% Triton X-100) at 4°C for 15 min. Samples were electrophoresed on 4% non-denaturing polyacrylamide gel at 50V and 4°C for 3h. (G) Graphical representation of results obtained from densitometry analysis of free DNA bands from gels of IFI16 binding with SS A50, DS NHEIII, Q NHEIII and cruciform DNA targets from three independent experiments with SD. Schemes of DNA structures in A-F are not to scale.

To compare IFI16 binding to quadruplex and double stranded DNAs derived from the HTEL sequence or from the NHEIII region of the *MYC* promoter, as well as to single stranded oligonucleotide and oligonucleotide forming a cruciform structure (CF), we used electrophoretic mobility shift assay with fluorescently labelled oligonucleotides described in Table 1. The quadruplex structures of G-rich single strands were formed by addition of 50 mM KCl and confirmed by CD spectroscopy (Fig 2A and 2B). The binding reactions were carried out in 15 μl reaction volumes with fixed DNA concentration (5 pmol for all DNA substrates) and increasing concentrations of IFI16 from 0 to 40 pmol. To stabilize the quadruplex structures, the EMSA was performed in the presence of 50 mM KCl in the gel as well as in the electrophoretic buffer. The same conditions were used also for other DNA substrates. As previously described by Morrone et al., double stranded DNA-IFI16 complexes do not penetrate into native PAGE gels due to the high pI of the protein (9.3) and oligomerization of IFI16 on double stranded DNA [25]. Therefore, we analyzed the reduction of free DNA signal intensities after IFI16-DNA binding (the intensities of the DNA bands are plotted in Fig 4G as fraction of bound DNA calculated from relative decrease of the band intensity).

We compared the preference of IFI16 to six different oligonucleotide targets (quadruplex HTEL, quadruplex NHEIII, double stranded HTEL and double stranded NHEIII, cruciform and single stranded DNA (Fig 4). IFI16 binding to quadruplex HTEL DNA leads to complete disappearance of the free DNA band at protein:DNA molar ratio 4:1 (Fig 4B, lane 6). In contrast, free double stranded DNA from the HTEL sequence (obtained by hybridization of the G-rich strand with the complementary strand) was visible up to a ratio of 8:1 (Fig 4A, lane 7). Similarly, when we studied the preference of IFI16 to quadruplex and double stranded DNA derived from the NHEIII region from the *MYC* promoter (Fig 4C and 4D), we observed complete disappearance of the free quadruplex DNA at protein:DNA molar ratio 4:1 (Fig 4D, lane 6). The comparison of binding to oligonucleotide targets derived from human genomic elements in double stranded forms on the one hand and quadruplex structures on the other provides evidence for a preference of IFI16 for quadruplex DNA.

Further, we tested IFI16 binding to single stranded and cruciform oligonucleotides to extend the comparative analysis of IFI16 protein binding to DNA adopting different structures. IFI16 protein binding to unstructured single stranded DNA was weak compared to other DNA targets (Fig 4E) as we observed a slight decrease of the free DNA band intensity only for the highest 8:1 protein:DNA ratio (Fig 4E, lane 7). Cruciform DNA (Fig 4F) was a more favorable target for IFI16 protein than single stranded and double stranded DNA and we observed a decrease in the signals of the free cruciform DNA at lower concentrations of IFI16 protein and 86% decrease of the free cruciform DNA band at the highest protein concentration (Fig 4F, lane 7). The most pronounced differences among the four DNA structures (6 oligonucleotides), reflecting different apparent affinities of IFI16 protein binding, were observed at protein:DNA ratio 4:1. At this ratio (20 pmol IFI16 in the reaction), only 5% of single stranded DNA, 50% of double stranded DNA (both double stranded HTEL and double stranded NHEIII) and 70% of cruciform was bound, but almost 90% of quadruplex DNA (both quadruplex HTEL and quadruplex NHEIII) was apparently in complex with IFI16. Densitometry analysis of three independent experiments is shown in Fig 4G. The trend of IFI16 to oligomerize on double stranded DNA molecules is visible as bound DNA “hanging” in the gel wells and not penetrating into the gel (Fig 4A, lane 5–7, Fig 4C, and lane 5–7). In the case of IFI16 binding to quadruplex (and CF) structures, we observed slightly smeared bands in the gel (Fig 4B, lane 6, 7, Fig 4D lane 6, 7, Fig 4F, lane 5, 6, 7). This can be explained by structure specificity, where the protein is able to recognize the structure of DNA and bind to DNA at lower protein concentrations, forming distinct, probably globular complexes capable of migrating in the gel, whereas filamentous complexes of double stranded DNA non-specifically bound by multiple proteins tend to form aggregates. Western blot analysis of the gels after EMSA (where we can see protein signals near the gel wells for double stranded DNA and smeared bands of IFI16 for quadruplex DNA binding) support this explanation (not shown). These results show that DNA structure is an important factor dictating IFI16 DNA binding preferences and that the non-B DNA structures, including cruciform structure and especially quadruplex DNA, are preferentially bound by IFI16 protein.

## Discussion

The DNA binding activity of IFI16 was studied in detail recently, confirming its DNA length dependent affinity [9, 25]. Studies of IFI16 interactions with supercoiled and cruciform DNA [26] show preferences for structurally constrained DNA. Other non B-DNA interactions of IFI16 have not yet been studied. Here, we performed a systematic study of IFI16 interactions with quadruplex DNA structures. These DNA structures, with growing evidence of their importance in biological processes, are highly abundant in human, bacterial and viral genomes. We focused on quadruplex forming sequences present in the human genome, specifically the quadruplex forming sequences from the NHEIII region of the *MYC* promoter forming a parallel quadruplex structure and from the HTEL sequence known to form an antiparallel quadruplex [48, 55–57].

In previous studies, full length IFI16 was reported not to bind single stranded DNA, but to interact with double stranded DNA in a sequence non-specific manner [25]. However, the HIN-A domain shows preference for G-rich single stranded DNA [22]. According to QGRS mapper [47], the G-rich sequence used in [22] is able to form a quadruplex structure. The similarity to RPA protein OB-fold supports our results of the quadruplex preferences, as RPA was shown to bind and unwind quadruplex DNA [58, 59]. In our study, we showed greater binding of full length IFI16 protein to quadruplex DNA arising from G-rich single stranded than to double stranded DNA from the same sequence. The results show very similar affinities for both of the quadruplex structures and similarly lower affinity to double stranded DNA. This indicates the general preferences of IFI16 for quadruplex DNA.

CD spectroscopy characterized the influence of IFI16 on quadruplex formation, where IFI16 enhanced the formation of quadruplex structures in low ion concentrations. The formation of different quadruplex structures suggests a general capacity for IFI16 to enhance quadruplex formation and stabilization. This feature was also described for other proteins with direct impact on the transcription pattern; for example, nucleolin enhanced the formation of quadruplex in the *MYC* promoter which led to a lower level of MYC protein [35]. The presence of IFI16 on the *MYC* promoter was shown by chromatin immunoprecipitation [60] and increased IFI16 expression had a negative effect on MYC levels, which led to a lower expression of hTERT [61].

The DNA length- and supercoil-dependency of IFI16 binding was described previously [25, 26]. To test the importance of quadruplex structure in the context of long DNA we used a 2959 bp-long plasmid DNA without quadruplex structure, its linear form and a supercoiled plasmid containing a 141 bp sequence from the NHEIII region of *MYC*. EMSA showed an evident preference for pCMYC; moreover, different band patterns obtained for the pBluescript and pCMYC plasmids indicated formation of a single, strongly preferred protein-DNA complex in the latter case. Thus, the quadruplex DNA structure is bound with preference not only in short oligonucleotides but also in long scDNA (i.e., under conditions which are closer to cells than oligonucleotides representing only the given DNA structure and completely omitting effects of long DNA stretches and their global topological state). The lack of apparent binding of IFI16 to linear plasmid DNA under the same conditions used for IFI16 binding to scDNA are in agreement with expectations (neither supercoils, nor open local structures such as the quadruplex DNA forms are present in unconstrained linear DNA).

H/D exchange has proven to be a powerful method for studying protein-protein interaction sites, where the interacting amino acids are identified by their protection from deuteration. Here we used this approach to identify the protected sites in IFI16 interacting with different forms of DNA. For the first time we have used H/D exchange as a method for the determination of protein-quadruplex interaction. IFI16 interaction with DNA at 1:1 molar ratio changed the deuteration profile when quadruplex DNA was bound. Both DNA binding domains (HIN-A and HIN-B) showed altered deuteration, representing strong interaction of these protein sites with quadruplex DNA. Single stranded and double stranded DNA did not induce such deuteration changes in the HIN domain. However, the PYRIN domain was influenced by all tested DNAs, in the order: single stranded DNA < double stranded DNA < quadruplex DNA. Given that the PYRIN domain is known to be involved in IFI16 oligomerization, these finding indicating that all of the DNA forms we tested may influence protein-protein interactions and perhaps subsequent oligomerization [25]. This result is in agreement with our EMSA results, where the presence of double stranded DNA caused oligomerization and protein binding to DNA, but the structure containing quadruplex DNA allowed more specific IFI16 binding. It has been shown that both HIN-A and HIN-B bind to single stranded and double stranded DNA [9, 22], however only short oligonucleotides without considering structural features of DNA was used in these studies. It was also shown that B-type or Z-type double stranded DNAs cause similar changes to the HIN-A domain [62]. Our comparison of IFI16 binding to single stranded, double stranded and quadruplex DNA showed a strong preference for quadruplex DNA binding. We observed the largest changes of IFI16 deuteration in complex with quadruplex DNA at amino acids 271–302 and 586–617 in HIN-A and HIN-B, respectively. This experiment also revealed new regions that might be responsible for the preferential binding of IFI16 to quadruplex DNA. In contrast to data from the crystal structure of HIN-B with ssDNA that forms only two polar contacts with DNA, our experiments showed that quadruplex DNA causes larger protection in this area and suggests involvement of other polar contacts. Surprisingly, all DNA forms induced changes in deuteration in the PYRIN domain, which is denoted as responsible for protein oligomerization. Our results point to involvement of the same parts of both HIN domains of IFI16 in binding to quadruplex DNA—where the linkers between the OB-fold and the OB-fold itself are involved in DNA binding.

## Conclusion

The importance of IFI16 in cell regulation and defence from pathogen infection is known. The exact mechanism(s) of IFI16 recognition of self and non-self DNA is not sufficiently described yet. Our study provides insight into the mechanism of IFI16-DNA interactions. Observations of IFI16 binding to quadruplex DNA in the context of long supercoiled DNA, as well as induction and stabilization of quadruplex structures, indicate possible mechanisms of IFI16’s action in regulation processes. Considering the activity of IFI16 in HIV-1 defence [20, 63, 64] and the discovery that interaction of nucleolin with a quadruplex structure in the HIV-1 LTR silences HIV-1 transcription [65], quadruplex recognition and stabilization by IFI16 is likely to be a crucial component of cellular viral defence mechanisms. The binding pattern differences using double stranded or quadruplex DNA also point out the importance of structure-specific binding in DNA recognition and oligomerization for the ability of IFI16 to selectively recognize and regulate cellular gene expression. Our findings provide a better understanding of IFI16 interactions and have implications for the biological functions and mechanisms of action of IFI16 in health and disease.
